# A decision algorithm to identify patients with high probability of monogenic diabetes due to HNF1A mutations

**DOI:** 10.1007/s12020-019-01863-7

**Published:** 2019-02-18

**Authors:** Magdalena Szopa, Tomasz Klupa, Maria Kapusta, Bartlomiej Matejko, Damian Ucieklak, Wojciech Glodzik, Barbara Zapala, Cyrus Maurice Sani, Jerzy Hohendorff, Maciej T. Malecki, Jan Skupien

**Affiliations:** 1 Department of Metabolic Diseases, Jagiellonian University Medical College, Krakow, Poland; 2 Department of Clinical Biochemistry, Jagiellonian University Medical College, Krakow, Poland; 3 Sanatio Medical Center, Krakow, Poland; 4 School of Medicine in English, Jagiellonian University Medical College, Krakow, Poland

**Keywords:** Biomarker, Monogenic diabetes, Decision tree, MODY

## Abstract

**Purpose:**

To investigate the utility of biomarkers of maturity-onset diabetes of the young (MODY), high-sensitivity C-reactive protein (hsCRP), and 1,5-anhydroglucitol (1,5-AG) in conjunction with other clinical and laboratory features to improve diagnostic accuracy and provide a diagnostic algorithm for HNF1A MODY.

**Methods:**

We examined 77 patients with HNF1A MODY, 88 with GCK MODY mutations, 99 with type 1 diabetes, and 92 with type 2 diabetes. In addition to 1,5-AG and hsCRP, we considered body mass index (BMI), fasting glucose, and fasting serum C-peptide as potential biomarkers. Logistic regression and receiver operating characteristic curves were used in marker evaluation.

**Results:**

Concentration of hsCRP was lowest in HNF1A MODY (0.51 mg/l) and highest in type 2 diabetes (1.33 mg/l). The level of 1,5-AG was lowest in type 1 diabetes and HNF1A MODY, 3.8 and 4.7 μg/ml, respectively, and highest (11.2 μg/ml) in GCK MODY. In the diagnostic algorithm, we first excluded patients with type 1 diabetes based on low C-peptide (C-statistic 0.98) before using high BMI and C-peptide to identify type 2 diabetes patients (C-statistic 0.92). Finally, 1,5-AG and hsCRP in conjunction yielded a C-statistic of 0.86 in discriminating HNF1A from GCK MODY. We correctly classified 92.9% of patients with type 1 diabetes, 84.8% with type 2 diabetes, 64.9% HNF1A MODY, and 52.3% GCK MODY patients.

**Conclusions:**

Plasma 1,5-AG and serum hsCRP do not discriminate sufficiently HNF1A MODY from common diabetes types, but could be potentially useful in prioritizing Sanger sequencing of *HNF1A* gene.

## Introduction

Maturity-onset diabetes of the young (MODY) is a heterogenic group of at least 13 single gene diseases characterized by autosomal dominant inheritance, diabetes onset in adolescents or young adults, and insulin deficiency with preserved insulin secretion [1, 2]. Their joint prevalence is estimated to be approximately between 0.5 and 2.0% of all diabetes cases in European populations [3]. In a pediatric population, ascertained through SWEET (Better control in Pediatric and Adolescent diabeteS: Working to crEate CEnTers of Reference) initiative its prevalence was 1.3% [4].

Genetic testing forms the basis for differentially diagnosing these forms of diabetes. Although no precise and uniform definition has been adopted, genetic testing is offered to patients meeting certain commonly accepted clinical criteria. An alternative to some fixed criteria is the probability calculator of MODY, which utilizes common clinical characteristics, such as family history, age, and age at diagnosis, sex, diabetes treatment regimen, body mass index (BMI), and glycated hemoglobin A_1c_ (HbA_1c_) [5]. Such a calculator estimates the probability of MODY in a patient without indicating the specific etiologic form of the disease.

The most common MODY types are HNF1A MODY and GCK MODY [6], with prevalence estimates varying widely between reports, primarily due to referral patterns in different countries and due to recruitment strategies. Widespread blood glucose screening in young, asymptomatic individuals leads to the identification of a higher proportion of GCK mutation carriers, while testing in symptomatic and risk group patients preferentially includes HNF1A MODY patients [3]. The report from SWEET database suggests that GCK MODY is the most prevalent, comprising approximately one-half of all MODY cases, wheseas HNF1A MODY is three times less frequent [4]. These two genes, *HNF1A* and *GCK*, have been the top targets in mutation search efforts employing Sanger sequencing. Recent advances in next-generation sequencing (NGS) allowed for targeting many more genes [7, 8], although availability of this method may still be limited. Nevertheless, NGS is economically feasible if samples are tested in large batches. Sanger sequencing remains a method of choice in smaller centers and for searching known mutations among family members of a proband. Prioritizing the analysis of specific genes based on a simple algorithm would potentially limit cost and workload issues in search of the most frequent MODY forms.

Several attempts have been made to distinguish between specific MODY types and type 1 or type 2 diabetes. The first suggested biomarker was 1,5-anhydroglucitol (1,5-AG), the decreased levels of which identified *HNF1A* mutation carriers [9]. This largely exogenous monosaccharide is reabsorbed in renal proximal tubules and achieves a steady concentration in serum. In hyperglycemia, glucose competes with it for reabsorption, increasing its urinary output, and reducing its serum level. In case of decreased renal threshold for glucose, 1,5-AG serum concentration decreases due to the similar mechanism. A second promising and well-studied marker was C-reactive protein assayed with a high-sensitivity method (hsCRP) [10–12]. Another biomarker, urinary C-peptide, serves rather as a discriminatory tool between any MODY diabetes and type 1 diabetes [13]. None of HNF1A MODY-related markers found wide utility in clinical practice. Indication for genetic testing for MODY is based on clinical criteria [14]. Simple and inexpensive criteria to distinguish GCK from HNF1A MODY using glycemic control parameters have been suggested [15].

The aim of this study was to investigate whether both HNF1A MODY biomarkers, hsCRP and 1,5-AG, together with other clinical and laboratory characteristics can improve diagnostic accuracy and provide a diagnostic algorithm of HNF1A MODY.

## Subjects and methods

In 2004, a database of MODY was initiated at the Department of Metabolic Diseases, Jagiellonian University Medical College in Krakow, Poland. Details of the inclusion and exclusion criteria have been published previously [9]. Briefly, we collected families with MODY phenotype defined as a three-generation autosomal dominant inheritance of diabetes, age at diagnosis under 25 years in at least two patients in the pedigree, and insulin independence (either over 1 year on diet therapy or oral drugs, or insulin dose <0.5 U/kg of body mass) of the proband. Almost 350 mutation carriers in MODY genes have been identified in our database so far. For this study, we contacted 205 adult mutation carriers in *HNF1A* or in *GCK* genes. Informed consent to participate in the study was received from 77 diabetic patients with HNF1A MODY and 88 GCK MODY mutation carriers, with either diabetes or prediabetes. In addition, we recruited 99 patients with type 1 diabetes and 92 patients with type 2 diabetes as consecutive case series. Type 1 diabetes was defined as diabetes with either acute onset ketoacidosis before 35 years of age or the presence of glutaminic acid decarboxylase autoantibodies and insulin dependence within 1 year from onset. Type 2 diabetes diagnosis was based on both clinical presentation and the presence of risk factors, without evidence suggesting monogenic, autosomal dominant etiology. We used the following exclusion criteria: pregnancy, liver cirrhosis, malignancy, steroid therapy, gastrectomy, and elevated serum creatinine level. The study protocol and informed consent procedures were approved by the Bioethical Committee of the Jagiellonian University and were concordant with the Declaration of Helsinki. Written informed consent was obtained from all study participants.

Blood samples were collected in fasting condition for biochemical evaluation. Serum and EDTA plasma were obtained by spinning whole blood specimens at 3500 rpm for 10 min, and were subsequently stored in −80 °C. 1,5-AG concentration was measured in EDTA plasma with 1,5-AG Elisa kit96T (Immuniq). Measurements of hsCRP were performed with hsCRP kit (ErbaMannheim) in serum. HbA_1c_ was measured in whole blood upon sample collection using high-performance liquid chromatography (Bio-Rad). Serum C-peptide concentration was measured with immunoassay using a Cobas 6000 analyzer (Roche Diagnostics). Diagnosis of hypertension was based on data in patients’ medical records. Diabetic retinopathy diagnosis was based on an ophthalmologic examination and albuminuria was defined as albumin/creatinine ratio >30 mg/g, as documented in medical records.

For statistical analysis, data were summarized as means and standard deviations, medians and quartiles, or percentages, where applicable. Levels of candidate biomarkers were compared between study groups using univariate and multiple regression models. Using logistic regression and receiver operating characteristic (ROC) analysis, we tested 1,5-AG and hsCRP singly and together for their ability to discriminate HNF1A MODY patients from the other patient groups: GCK MODY, type 1 diabetes, and type 2 diabetes. C-statistic was used as a discrimination metric.

In building a MODY diagnostic algorithm, in addition to 1,5-AG and hsCRP, we considered BMI, fasting glucose, and serum C-peptide as potential disease markers. To build a diagnostic algorithm, we sought a single marker or a set of statistically significant markers, which best distinguished any one of the four study groups (HNF1A MODY, GCK MODY, type 1 diabetes, type 2 diabetes) from the remaining three together. Sets of candidate markers were built by adding sequentially additional variables to a model with the single best (in terms of C-statistic) marker. Additional markers were retained if their inclusion significantly (*p* < 0.05) improved the C-statistic. After the best marker set was established, the study group identified by this marker set was excluded and the procedure was repeated with the remaining three groups, and then with the remaining two groups. This produced a sequential, three-step algorithm in the form of a decision tree. Statistical analysis was performed in SAS 9.4 software and R 3.4.4. *p*-Values < 0.05 were considered significant.

## Results

Characteristics of the study groups are provided in Table 1. The patients with type 2 diabetes had the shortest diabetes duration and were the oldest at diabetes diagnosis. Individuals with GCK MODY were the youngest when first diagnosed with diabetes or impaired fasting glucose. Patients with type 2 diabetes had the highest BMI and serum creatinine, wheseas BMI and creatinine were similar in the three other groups. All patient groups were characterized by good glycemic control, with the highest HbA_1c_ and fasting glucose levels in patients with type 1 diabetes. Fasting glucose was the lowest in both MODY groups. Hypertension was present in over 20% of patients in MODY and type 1 diabetes groups, while its prevalence reached 80% in patients with type 2 diabetes. GCK MODY patents were virtually free from diabetes complications. Patients with type 2 diabetes were predominantly treated with non-insulin drugs. In 77% of GCK MODY patients, diabetes was controlled with diet only. In HNF1A MODY, insulin was prescribed in 35% and non-insulin agents in 48%. All type 1 diabetes patients were treated with insulin.

**Table 1:** Characteristics of the study groups

Group	HNF1A	GCK	T1D	T2D
*N*	77	88	99	92
Females (%)	64.9	61.4	39.1	61.6
Age (years)	38.8 ± 15.2	34.7 ± 15.2	29.1 ± 10.3	59.4 ± 10.2
Years from disease diagnosis	17.3 ± 10.2	26.2 ± 12.4	12.5 ± 8.0	6.5 ± 6.5
Age at disease diagnosis (years)	23.1 ± 12.1	7.3 ± 7.3	16.6 ± 9.0	52.9 ± 10.7
BMI (kg/m^2^)	23.9 ± 4.2	23.7 ± 4.6	23.9 ± 2.7	30.3 ± 4.6
Serum creatinine (μmol/l)	75.5 ± 15.9	74.0 ± 13.4	74.4 ± 13.4	82.7 ± 20.8
HbA_1c_ (%)	6.9 ± 1.4	6.3 ± 0.7	7.4 ± 1.1	6.8 ± 1.2
(mmol/mol)	51.9 ± 15.3	45.4 ± 7.6	57.4 ± 12.0	50.8 ± 13.1
Treatment				
with insulin (%)	35	6	100	17
with non-insulin agents (%)	48	17	0	81
with diet only (%)	17	77	0	2
Fasting glucose (mmol/l)	6.8 ± 2.6	6.9 ± 1.3	8.1 ± 3.0	7.6 ± 1.9
Hypertension (%)	22.1	21.6	23.2	80.4
Diabetic retinopathy (%)	16.9	0	12.1	7.6
Albuminuria (%)	9.1	0	3.0	5.4

Data are mean ± standard deviation or percentages

*BMI* body mass index, *GCK* GCK MODY, *HNF1A* HNF1A MODY, *T1D* type 1 diabetes, *T2D* type 2 diabetes, *MODY* maturity-onset diabetes of the young, *HbA*_*1c*_ glycated hemoglobin A_1c_

Concentrations of the most important candidate biomarkers are presented in Table 2. As expected, the lowest level of hsCRP was observed in HNF1A MODY patients. In contrast, hsCRP level was highest in the type 2 diabetes group. Serum concentration of 1,5-AG was the lowest in type 1 diabetes group, followed by HNF1A MODY and type 2 diabetes patients. The highest concentration of 1,5-AG was in GCK MODY patients. Serum C-peptide was undetectable in most patients with type 1 diabetes. Its level was the highest in type 2 diabetes, wheseas no notable differences were present between the two MODY groups. Adjusting for potential confounders, including sex, age (or diabetes duration), BMI, HbA_1c_, and serum creatinine level did not significantly alter the relationships between study groups in 1,5-AG levels or C-peptide levels. 1,5-AG concentration remained the highest in GCK MODY. It was significantly lower in type 2 diabetes group (*p* < 0.001 vs. GCK MODY), and the lowest in type 1 diabetes and HNF1A MODY patients (*p* < 0.001 and *p* = 0.017, respectively vs. type 2 diabetes). The adjusted difference between HNF1A MODY and type 1 diabetes did not reach statistical significance (1.1 μg/ml, *p* = 0.14). No relationship was found between diabetes treatment modalities and 1,5-AG. Serum C-peptide in adjusted analysis remained the lowest in type 1 diabetes and the highest in type 2 diabetes (*p* < 0.001 in all pairwise comparisons), wheseas there was no significant difference between HNF1A and GCK MODY (*p* = 0.22). After adjusting for confounders, the level of hsCRP remained lower in HNF1A MODY than in GCK MODY (*p* = 0.033) or in type 1 diabetes (*p* = 0.018), wheseas the adjusted difference between type 2 diabetes and HNF1A MODY was not significant, although it remained numerically large (0.5 mg/l, *p* = 0.45).

**Table 2:** Concentrations of plasma/serum biomarkers of MODY

Group	HNF1A	GCK	T1DM	T2DM	*p*-Value
hsCRP (mg/l)	0.51 (0.46, 0.66)	0.76 (0.52, 1.55)	0.75 (0.53, 1.41)	1.33 (0.77, 2.50)	<0.001
1,5-AG (μg/ml)	4.70 (2.78, 8.19)	11.24 (6.54, 14.28)	3.77 (1.94, 6.02)	6.07 (3.75, 10.54)	<0.001
C-peptide (ng/ml)	1.45 (1.07, 1.80)	1.43 (0.96, 1.80)	0.01 (0.01, 0.10)	2.78 (1.95, 3.55)	<0.001

*GCK* GCK MODY, *HNF1A* HNF1A MODY, *T1D* type 1 diabetes, *T2D* type 2 diabetes, *hsCRP* high-sensitivity C-reactive protein, *1,5-AG* 1,5-anhydroglucitol, *MODY* maturity-onset diabetes of the young

Significant overlaps of the distributions of markers between the study groups suggest that no single marker can identify MODY patients and distinguish them from the common diabetes types. The best marker of HNF1A MODY in the total study cohort was hsCRP. It was characterized by the C-statistic (with 95% confidence interval) only 0.732 (0.675, 0.789). The C-statistic of 1,5-AG was merely 0.611 (0.545, 0.676) in the four study groups combined. Its addition to hsCRP failed to improve discrimination accuracy.

**Fig. 1:**
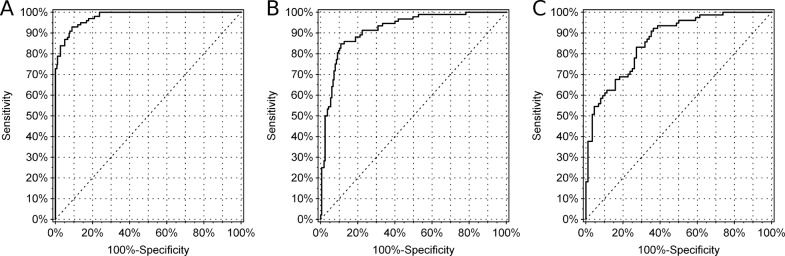
Receiver operating characteristic (ROC) curves for the fasting serum C-peptide discriminating type 1 diabetes from type 2 diabetes, GCK maturity-onset diabetes of the young (MODY) and HNF1A MODY together (**a**), for fasting serum C-peptide and body mass iindex (BMI) to distinguish type 2 diabetes from GCK and HNF1A MODY (**b**) and for 1,5-anhydroglucitol (1,5-AG) with high-sensitivity C-reactive protein (hsCRP) to discriminate HNF1A MODY and GCK MODY(**c**)

We decided to develop a multi-step clinical decision tree that uses various clinical characteristics and biomarkers to identify patients with MODY. In the first step, we sought a marker or a set of markers that would identify one of the four study groups. The best marker in this step was serum C-peptide singly, which identified patients with type 1 diabetes with a C-statistic of 0.976 (95% confidence interval 0.962, 0.989). The ROC plot is presented in Fig. 1a. A threshold of 0.6 ng/ml identified patients with type 1 diabetes with 92.9% sensitivity and 91.0% specificity. In the next step, we identified two markers of type 2 diabetes: C-peptide and BMI. Their joint discrimination (C-statistic) of type 2 diabetes patients from the two MODY groups was 0.917 (0.881, 0.952), as shown in Fig. 1b. We created an index from these two variables, weighted by their regression parameters: C-peptide [ng/ml] + 0.16 × BMI [kg/m^2^]. A threshold 6.29 in this index was characterized by 84.8% sensitivity and 89.1% specificity. In the final step, we sought markers best distinguishing HNF1A and GCK MODY. We identified 1,5-AG and hsCRP, which jointly resulted in a C-statistic of 0.863 (0.809, 0.917), see Fig. 1c. The index created from these two variables was in the following form: 1,5-AG [μg/ml] + 1.56 × hsCRP [mg/l]. A threshold 10.16 was characterized by 83.1% sensitivity and 72.7% specificity in detecting HNF1A mutation carriers. At this step, the strongest single marker discriminating HNF1A MODY vs. GCK MODY was 1,5-AG, and its C-statistic reached 0.831 (0.771, 0.891). In this setting, C-statistic of hsCRP was 0.686 (0.604, 0.768).

**Fig. 2:**
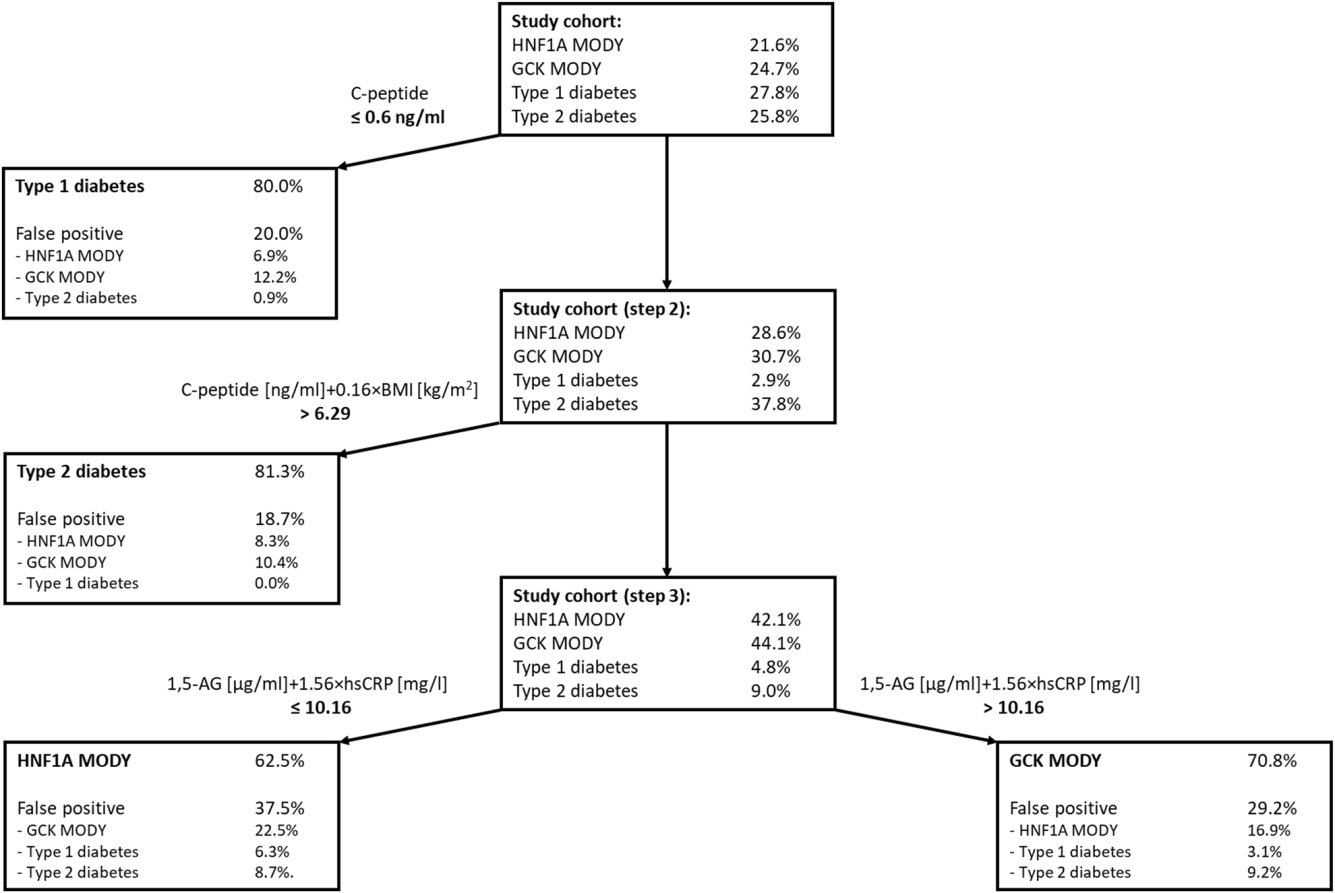
Application of the three-step decision tree algorithm to identify patients with HNF1A maturity-onset diabetes of the young (MODY) showing true-positive and false-positive rates in the tree branches

To simulate the performance of the three-step algorithm in practice we applied it to the entire study cohort, retaining misclassified patients at each step. As a result, the algorithm correctly identified 92/99 (92.9%) of patients with type 1 diabetes, 78/92 (84.8%) patients with type 2 diabetes, 50/77 (64.9%) HNF1A MODY patients, and 46/88 (52.3%) of GCK mutation carriers. The results of applying the algorithm to the study cohort are shown in Fig. 2.

## Discussion

In this study, for the first time we test together two postulated biomarkers of HNF1A MODY, 1,5-AG and hsCRP. Both markers do not perform well in distinguishing this monogenic disease from common diabetes types. Our previous publication demonstrated that 1,5-AG may distinguish HNF1A MODY from type 2 diabetes within HbA_1c_ range 6.5–9.0% [8]. A subsequent replication of our finding revealed that 1.5-AG in serum could not distinguish between autoimmune diabetes and HNF1A MODY, wheseas it performed well in differential diagnosis from GCK MODY [16], in line with our current findings. In this replication study [16], the diagnostic differential performance with respect to type 2 diabetes was not satisfactory even after adjusting for HbA_1c_.

The second possible marker, hsCRP was characterized by a better diagnostic performance. Its serum level was lower in HNF1A MODY when compared with GCK MODY, type 1, and type 2 diabetes [10]. In another study in patients with different MODY types, hsCRP level was lowest in *HNF1A* mutations carriers [11]. A subsequent multicenter study confirmed its satisfactory performance as an HNF1A MODY biomarker [12], while recently published results suggest its inferior utility among patients with antibody-negative diabetes [17].

In line with previous findings, we observed the lowest concentration of hsCRP in HNF1A MODY patients. There was, however, a significant overlap of its distribution with both *GCK* mutation carriers and type 1 diabetes groups. The largest contrast was seen with type 2 diabetes patients; however, it was much diminished with adjustment for the differences in age and BMI. Patients with type 2 diabetes were best identified by high BMI and high fasting serum C-peptide. Addition of hsCRP at this step did not improve the diagnostic accuracy of our algorithm. The other biomarker, 1,5-AG, had the lowest plasma concentration in type 1 diabetes, not in HNF1A MODY group. The likely explanation is that hyperglycemic excursions are the highest and most frequent in patients with type 1 diabetes, which depletes the pool of 1,5-AG in blood [18, 19] even in the absence of markedly lowered renal threshold for glucose [20]. In HNF1A MODY, lowered renal threshold for glucose [21–23] and milder than in type 1 diabetes postprandial hyperglycemia [24] (note the highest HbA_1c_ in type 1 diabetes group) exert opposite effects on 1,5-AG level.

Our findings indicate that the two markers of HNF1A MODY could only be applied to distinguish this form of monogenic diabetes from other MODY types, but neither from type 1 nor type 2 diabetes. Based on our results, we suggest that in clinical practice patients with type 1 diabetes may be excluded from candidates for genetic testing for MODY using fasting serum C-peptide or urinary C-peptide/creatinine ratio. This should be done in conjunction with autoantibody testing, especially in individuals with a latent course of disease and preserved insulin secretion. Interestingly, our data indicate that type 2 diabetes patients can be distinguished from MODY patients using clinical features, such as obesity and hyperinsulinemia. Such basic characteristics perform better than the biomarkers, hsCRP and 1,5-AG. Although metabolic syndrome may independently coexist with monogenic diabetes, MODY mutation carriers seem to be characterized by relatively more pronounced insulin secretion defect, hence good diagnostic performance of combination of BMI with fasting serum C-peptide. Our data suggest that fasting serum C-peptide <2.0 ng/ml indicate HNF1A MODY or GCK MODY with 84–87% sensitivity.

Our suggested algorithm correctly identified only 65% of HNF1A MODY patients, and 52% of *GCK* mutation carriers. The first two steps of the algorithm were characterized by an excellent discrimination (C-statistic 0.98 and 0.92), comparable to the reported discrimination of much more complex MODY probability calculator (0.95 and 0.98 for discrimination from type 1 and type 2 diabetes, respectively) [5]. Despite that, 24% of MODY patients in our study were misclassified as having either type 1 or type 2 diabetes. Optimizing thresholds to lower misclassification rate of MODY would result in increased misclassification of type 1 and type 2 diabetes into MODY groups and decreased positive predictive value of the algorithm. Our diagnostic algorithm is neither specific, nor sensitive enough to allow for identification of HNF1A MODY patients without gene sequencing and it cannot be used to make a clinical diagnosis of HNF1A or GCK MODY.

Possible practical utility of the biomarkers, 1,5-AG and hsCRP, can be considered only in the context of defining a group of patients with high probability of HNF1A MODY. Although high-throughput DNA sequencing renders clinical identification of MODY types a lesser priority [7], we would like to emphasize that Sanger sequencing is still widely used in smaller centers, with insufficient volume of referrals to make NGS economically feasible. In such cases, prioritizing *HNF1A* sequencing in some probands using 1,5-AG (an approximate cost of 1,5-AG assay is €9 per sample) in combination with hsCRP is an option worth considering in future research. Similarly, prioritizing *GCK* or *HNF1B* sequencing should be considered in probands, who present distinct clinical features of these MODY forms [14]. Only those patients with uncertain diagnosis of MODY type and with negative targeted sequencing results would be further referred to a larger center for NGS of a complete panel of monogenic diabetes genes.

This study has a few shortcomings we would like to acknowledge. First, we did not include patients with less frequent MODY forms and did not test the biomarkers in them, due to limited number of probands with confirmed disease and expected lack of statistical power to reach conclusive findings. Second, definition of our study groups resulted in a significant imbalance in age, BMI, diabetes duration, and treatment regimens, however, the proposed algorithm was not affected by adjusting for these confounders. Also, treatment modalities do not seem to affect the levels of biomarkers, including 1,5-AG [22]. Third, for excluding type 1 diabetes patients, we did not test for islet autoantibodies. We used fasting serum C-peptide instead of C-peptide/creatinine ratio in urine [13], as urine samples were not available from every study participant. Despite that, fasting serum C-peptide was characterized by an excellent performance in identification of patients with type 1 diabetes. In addition, one should consider replicating the performance of the algorithm in patients with incident diabetes, where it would be more clinically applicable. Finally, practical utility and cost-effectiveness of suggested application of HNF1A MODY biomarkers to qualify patients for Sanger sequencing requires an independent replication.
